# Novel euthanasia technique for zebrafish using electric shock in standard group housing aquaria

**DOI:** 10.1038/s41598-025-87540-4

**Published:** 2025-01-23

**Authors:** Ulla Saarinen, Erika Sundell, Lynne Sneddon, Albin Gräns

**Affiliations:** 1 Department of Applied Animal Science and Welfare, Swedish University of Agricultural Sciences, Gothenburg, Sweden; 2 Department of Biological & Environmental Sciences, University of Gothenburg, Gothenburg, Sweden

**Keywords:** *Danio rerio*, Humane killing, Stunning, Animal welfare, Electrocution, Gill ventilation, Animal behaviour, Animal physiology

## Abstract

Zebrafish are widely used in research, accounting for approximately 50% of all laboratory fish across Europe. Due to their rapid reproduction and aging, effective and practical euthanasia methods are necessary for managing large stocks. This study investigated whether adult zebrafish can be euthanised by delivering an electrical current directly via the water in their housing aquaria. For this, portable plate electrodes were developed for 3.5 and 8.0 L Tecniplast™ housing aquaria. Our results show that adult zebrafish can be euthanised in groups (5 fish per litre) with electrical fields and current densities of at least 6 or 7 V_RMS_ cm^−1^, and 0.7 or 0.8 A_RMS_ dm^−2^ in 3.5–8 L aquaria, respectively, when delivered for 30 s in water at 28 °C with a conductivity of 800 µS cm^−1^ at 50 Hz. If widely implemented, this technique could improve the welfare of the vast number of zebrafish used across the world.

**Supplementary Information:**

The online version contains supplementary material available at 10.1038/s41598-025-87540-4.

## Introduction

The use of the zebrafish (D*anio rerio*, F. Hamilton 1822) as a model species in research has rapidly increased over the past few decades due to their versatile research applications and practical advantages^1–6^. Managing large stocks of zebrafish strains requires effective practices, including euthanising (i.e. the act of killing an animal humanely) older fish to make space for younger ones. Removing older fish can also benefit the health and welfare younger fish by reducing overcrowding and disease transmission^7^. Although zebrafish can live up to 2–3 years, they typically show signs of aging and pose a health risk to themselves and the colony from around 12 months of age^8^. Therefore, there is a pressing need for an accessible and effective method to euthanise zebrafish. Two common methods for euthanising batches of zebrafish today are (i) an overdose of a water-soluble anaesthetics and (ii) live-chilling in cold water followed by brain destruction or exsanguination^9–11^. However, the humaneness of these methods for zebrafish have been questioned, as these approaches may be aversive. Both methods require capturing, handling and transferring the fish out of their housing aquaria, procedures that are stressful and can compromise their welfare^14^ For example, the anaesthetics Tricaine (MS-222) and benzocaine often induce adverse behaviours such as twitching, erratic swimming and avoidance in zebrafish^12–16^. Additionally, the use of expensive and sometimes toxic anaesthetic chemicals raise environmental and economic concerns.

In aquaculture, electricity is recognized for its potential to stun (render unconscious) fish humanely and effectively during slaughter, as it can induce an immediate loss of consciousness^17–21^. However, applying electricity to farmed species intended for human consumption can be problematic due to high intensity shocks and/or prolonged exposure times compromising product quality^17,21–23^. Conversely, these concerns are not important when euthanising research animals, making high intensity electrical shocks that ensures death a viable option in laboratories. Currently, little is known about the use of electricity for euthanising fish used in research. Some evidence supports its efficacy with zebrafish embryos and larvae, but information on its applications for adult zebrafish is limited^24,25^. There is, however, a study where juvenile salmonids similar to the size of adult zebrafish were successfully euthanised using in-water electricity^26^. The main barriers for adopting electrical euthanasia in laboratory settings likely stem from the lack of suitable equipment and inadequate methods, safety concerns for using electricity, and established habits favouring anesthetic methods^11,12^.

Here, we developed and validated a novel method for euthanising adult zebrafish housed in laboratory conditions using in-water electric shock. Our specific aims were to: (i) determine if adult zebrafish can be immediately stunned and euthanised with the use of an electrical shock, and (ii) assess whether this method can be used for groups of zebrafish housed in commercial Tecniplast™ aquaria at recommended stocking densities. We hypothesized that the success rate of electro-euthanasia (i.e., when zebrafish do not recover following the electrical shock) would rise with increased intensity and/or duration of the electrical shock.

## Materials and methods

### Ethics statement

All experiments, methods, handling and housing was carried out in accordance with Swedish (Animal protection act, 2018:1192) and European (EU directive, 2010/63/EU) guidelines and regulations for the care and use of laboratory animals, and approved by Regional Animal Ethics Committee in Gothenburg (permit 5.8.18–16941/2021). All animal experimental procedures were performed and are reported in accordance with ARRIVE guidelines^27^.

### Animals and housing

A total of 650 adult zebrafish (462 ± 7 mg) was used in this study. All fish used were scheduled to be euthanised by colony management due to old age. Two strains of zebrafish, AB and C (CaMPARI: calcium-modulated photoactivatable ratiometric integrator), were included in the study^28^. The AB fish were obtained from the Zebrafish Core Facility at the Karolinska Institute in Stockholm, Sweden, at 4 months of age. They were then transported to the University of Gothenburg, where they were housed in the animal facility for 14 months before the current study. The C strain originated from University College London, United Kingdom, and the line was maintained at the University of Gothenburg since November 2020. The fish derived from this line used in this study were 14–18 months old. Both strains were kept at the recommended stocking densities of 5 fish L^− 1^, at a 14:10 h light: dark cycle and were fed *ad libitum* with granules (Tetra GmbH, Melle, Germany) twice per day and with live *Artemia* once per week^29^. Zebrafish were housed on a Tecniplast recirculation racking system in 3.5–8 L Tecniplast™ aquaria (Tecniplast S.p.A., Buguggiate, Italy) with internal mechanical and biological filtration in a sump tank^30^. Males and females were housed together at an approximately equal sex ratio. The water temperature was monitored daily and maintained at 28 ± 1 °C. The water conductivity was checked weekly using an AP-2 digital water tester (HM Digital Inc., Carson, California, U.S.A), and adjusted to 700 ± 100 µS cm^− 1^ by adding Tetra Marine SeaSalt (Tetra GmbH, Melle, Germany) to distilled water if needed. Water quality parameters including pH, ammonia, nitrate, and nitrite were tested weekly using the Aquatest Combiset Marin (JBL GmbH & Co.KG, Neuhofen, Germany). pH was kept at 7.4, and ammonia, nitrite and nitrate were maintained below 0.1, 0.1, and 10 mg/l, respectively by the biological filter and by a daily 10% water change.

### Setup for the electrical euthanasia

All electrical euthanasia experiments were conducted in a controlled and isolated experimental room separated from the zebrafish housing systems located in other rooms.

**Fig. 1:**
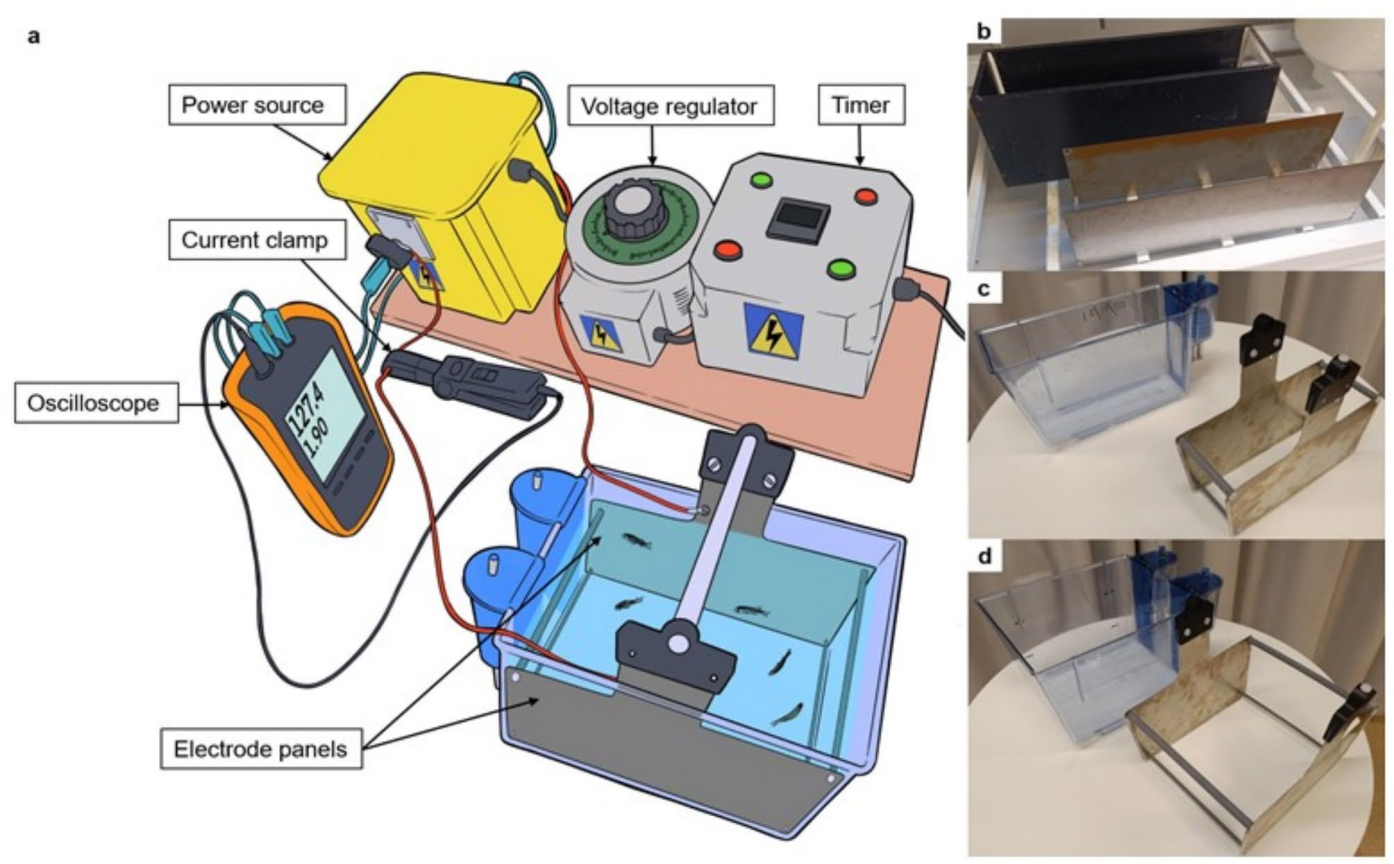
Illustration of the electrical euthanasia setup (**a**) and photos of the experimental tank and aquaria and corresponding electrodes used (**b**-**d**). The intensity and duration of the electrical shocks were controlled using a voltage regulator and timer (a). Panel b shows the experimental tank along with its corresponding electrode panels used in initial trials. Panels c and d display Tecniplast aquaria with custom-made electrode panels: c shows the 3.5 L aquarium and d shows the 8 L aquarium. Both housing aquaria accommodate identical electrode panels, adjustable to fit each aquarium by altering the length of the supporting rods (i.e., the only dimensional difference between the aquaria is the width). Silicone tubing was fitted along the edges of the electrodes panels to prevent damage to the aquarium walls. Additionally, the panels were equipped with a removable plastic handle at the top for easy placement (a).

The electrical euthanasia setup was comprised of a custom-built device assembled by Ace Aquatec (Ace Aquatec Ltd. Dundee, United Kingdom) connected to two stainless steel plate electrodes measuring 470 × 1 × 150 mm (L x W x H) spaced 110 mm apart in a 7.2 L experimental tank (Fig. 1a-b). The device included a variable AC transformer connected to an isolating transformer capable of delivering 50 Hz sinusoidal wave AC from 0 to approximately 250 root-mean-square voltages (V_RMS_). A low-frequency sinusoidal alternating current (AC) was selected for this study, as it has been demonstrated to achieve reliable stunning in fish^31^. A timer connected to the variable AC transformer’s power supply was used to control the exposure duration. Voltage and current during the electrical exposures were monitored using an oscilloscope. (Fluke 123B Industrial Scope meter, Fluke Europe B.V., Eindhoven, Netherlands) and a current clamp (Fluke, model 80i-110s).

After conducting the initial trials (see section Validation of electrical exposure as a mean to humanely stun and euthanise zebrafish) with the experimental tank (Fig. 1b) to assess immediate stunning and euthanasia potential of electrical exposure on zebrafish, new custom-made stainless steel panel electrodes (235 × 1 × 123 mm, L x W x H) were fabricated. These electrodes were designed to fit into standardized Tecniplast aquaria, commonly used for housing zebrafish in research facilities. The transition from the experimental tank to the Tecniplast aquaria aimed to develop and validate a prototype for portable electrodes suitable for use in laboratory facilities housing zebrafish in large numbers. Tecniplast aquaria were available in two sizes: 3.5 and 8 L, both sharing sidewall dimensions (i.e., differing only in width). Therefore, supporting rods positioned between the electrode panels were manufactured in two lengths to accommodate the two different widths, 94 mm for the 3.5 L aquarium and 210 mm for the 8 L aquarium. The experimental setup with the two aquaria containing the electrode panels is illustrated in Fig. 1c-d.

After each trial, the water in the experimental tank/aquaria was replaces to minimise the possibility of residual substances from the fish such as stress hormones, pheromones, and metabolic waste remaining in the water and potentially influence the outcomes of subsequent trials^32–34^. Tap water, aerated and left for at least 24 h to allow for chlorine evaporation, was used in all trials. Water temperature and conductivity were monitored using a HQd Portable Meter (HACH Lange GmbH, Loveland, Colorado, U.S.A), and held constant at 28 ± 0.9 °C and 800 ± 14 µScm^− 1^, respectively. These conditions were consistent with the standard zebrafish housing conditions in our laboratory.

Throughout all experimental trials, aquaria were filled to fully submerge the electrode panels. To achieve varying electrical field strengths and current densities, the output voltage of the electrical device was adjusted (details of the electrical parameters are outlined in Table 1). A Logitech C922 Full HD 1080P Pro camera (Logitech Europe S.A., Ecublens, Switzerland) was positioned above the experimental tank/aquaria to document all the trials. The camera footage was recorded using WebCam Monitor software version 1.4.0.0.

**Table 1:** Summary of the electrical trials conducted in the 7.2 L experimental tank, and the 3.5 and 8 L tecniplast housing aquaria.

Tank/aquarium (L)	No .trials	Fish trial^− 1^	Duration (s)	Field strength (V_RMS_ cm^− 1^)	Current density (A_RMS_ dm^− 2^)	Success rate (%)	Strain (AB/C)
7.2	2	5	1	4 ± 0.02	0.4 ± 0.001	80	10/0
7.2	2	5	1	5 ± 0.02	0.5 ± 0.003	100	10/0
7.2	2	5	30	5 ± 0.02	0.5 ± 0.001	100	10/0
3.5	11	10	30	5 ± 0.02	0.6 ± 0.003	100	60/50
3.5	1	15	30	5	0.7	93	0/15
3.5	5	15	30	6 ± 0.01	0.7 ± 0.009	100	30/60
8	4	10	30	5 ± 0.01	0.5 ± 0.006	90	10/30
8	6	10	30	6 ± 0.03	0.7 ± 0.007	100	60/0
8	2	40	30	6 ± 0.01	0.7 ± 0.007	98	45/35
8	6	40	30	7 ± 0.02	0.8 ± 0.01	100	160/80

The electrical shock durations were 1–30 s. short shocks were used to determine if the effect was immediate and were deemed successful if no signs of aversive behaviours were observed. Longer shocks as were used to investigate if the method for euthanising adult zebrafish under laboratory conditions were effective, i.e., if no signs of recovery were observed within 30 min after electrocution. All trials were conducted with a water temperature maintained at 28 ± 0.9 °C and a conductivity maintained at 800 ± 14 µScm^− 1^. Data are reported as means ± s.e.m.

### Electrical euthanasia trials

#### General outline of the protocol

See the detailed safety plan for humans in Supplementary Material.

All experiments were conducted between September 2022 and May 2023 between 8 am and 5 pm. Prior to each trial, electrical voltage was applied without fish in the water and adjusted to achieve the current and voltage that was intended for the trial. The number for fish that were planned for the trial were then transferred from their housing aquaria to the experimental tank or Tecniplast aquarium shortly before each trial at a time to minimize the stress of changing environment. Following the electrical shock, the fish remained in the experimental set up for observation until signs of recovery were displayed or for at least 30 min if no recovery was observed. Fish showing signs of recovery were promptly euthanised with an overdose (250 mg L^− 1^) of MS-222 (Sigma-Aldrich, Sain Louis, Missouri, U.S.A). Visual indicators of recovery including gill movements, body movements such as swimming, coordinated fin movements, hovering or flexing, response to stimuli and ability to maintain or regain equilibrium were inspected during and following the electrical shock. Delayed convulsions (muscle spasms) were not considered signs of recovery, as this has not been recognised as such previously^35^. Confirmation of recovery status was based on video recordings analysed in details after the trials.

#### Validation of electrical exposure as a mean to humanely stun and euthanise zebrafish

The initial trials in the experimental tank aimed to determine the electric field strength and current density required to stun the zebrafish promptly (i.e., within 1 s) without causing aversive behaviours indicative of stress. The electrical shock duration was limited to 1 s, because if longer electrical shock durations are tested without assessing short shock durations, there is a risk that any observed effects such as permanent loss of consciousness or death are caused by muscle exhaustion^20^. Brief electrical exposures, which only induce transient loss of consciousness, are crucial to study immediate effects of electrical shocks^20^. Fish that remained partially mobile following the electrical shock or exhibited aversive behaviours, such as increased swimming, hyperventilation, escape reactions or hiding behaviours shortly after the shock, were considered unsuccessfully stunned. When this occurred, the electric field strength was increased by 1 V_RMS_ cm^− 1^ before continuing with the next trial until a success rate of 100% (no fish showed aversive behaviour) was achieved. Once the minimum electric field strength that resulted in 100% success rate was found, the shock duration was prolonged to 30 s in the following trials to investigate the potential of the electric shock for euthanasia.

#### Validation of the use of portable electrodes fit for standardized zebrafish housing aquaria as a method of euthanasia

After visual confirmation in the experimental tank that (i) the 1 s electrical shock induced immediate loss of consciousness and (ii) a prolonged shock duration of 30 s resulted in euthanasia of the zebrafish, trials were conducted using the custom-made electrode panels designed to fit the Tecniplast zebrafish aquaria. Initially, trials where the shock lasted 30 s were performed with groups of 10 fish in both the 3.5 L and 8 L aquaria. This group size allowed for effective visual assessment during and after the electrical shock. Subsequently, the number of fish per trial was increased to the maximum capacity of the housing aquaria, accommodating 15 fish in the 3.5 L and 40 fish in the 8 L aquaria. This maximum capacity was determined based on the recommendation to rear zebrafish at a density of 5 fish L^− 1 29^. If not all fish in the aquaria were successfully euthanised (i.e., recovered within 30 min after the electrical shock), the electric field strength was increased by 1 V_RMS_ cm^− 1^ before continuing with the next trial until a success rate of 100% (no fish recovered during the 30 s shock or 30 min observation period) was achieved. One of the successful trials conducted in the 8 L aquarium can be inspected in Supplementary Movie 1.

### Data collection and processing

For each trial, the voltage output, current delivered though the water, and the duration of the shock were documented. Upon completion of each trial, the weight of each individual was recorded. The number of fish per trial and number of trials can be seen in Table 1. Electric fields and current densities were calculated using Eqs. (1) and (2), respectively:

1 $$\:\text{E}\text{l}\text{e}\text{c}\text{t}\text{r}\text{i}\text{c}\:\text{f}\text{i}\text{e}\text{l}\text{d}\:\text{s}\text{t}\text{r}\text{e}\text{n}\text{g}\text{t}\text{h}\:\left({\text{V}}_{\text{R}\text{M}\text{S}}{\text{c}\text{m}}^{-1}\right)=\frac{\text{V}\text{o}\text{l}\text{t}\text{a}\text{g}\text{e}\:\left({\text{V}}_{\text{R}\text{M}\text{S}}\right)}{\text{E}\text{l}\text{e}\text{c}\text{t}\text{r}\text{o}\text{d}\text{e}\:\text{s}\text{e}\text{p}\text{a}\text{r}\text{a}\text{t}\text{i}\text{o}\text{n}\:\left(\text{c}\text{m}\right)}$$

2 $$\:\text{C}\text{u}\text{r}\text{r}\text{e}\text{n}\text{t}\:\text{d}\text{e}\text{n}\text{s}\text{i}\text{t}\text{y}\:\left({\text{A}}_{\text{R}\text{M}\text{S}}{\text{d}\text{m}}^{-2}\right)=\:\frac{\text{C}\text{u}\text{r}\text{r}\text{e}\text{n}\text{t}\:\left({\text{A}}_{\text{R}\text{M}\text{S}}\right)}{\text{E}\text{l}\text{e}\text{c}\text{t}\text{r}\text{o}\text{d}\text{e}\:\text{a}\text{r}\text{e}\text{a}\:\left({\text{d}\text{m}}^{2}\right)}$$

## Results and discussion

On rare occasions, individual fish evaded the electrical shock by hiding between the tank or aquarium wall and electrode panel. These incidents occurred three times during the study, and the affected individuals were omitted from the results. Among the few fish that recovered from electrical shock, no differences were observed between the two strains; fish from both the AB and C strains appeared to respond similarly to the electrical shocks (Table 1).

### Minimum electrical intensity needed to stun and euthanise zebrafish in the experimental tank

Initial trials where a 1 s shock duration was applied in the 7.2 L experimental tank revealed that an electric field strength of at least 5 ± 0.02 V_RMS_ cm^− 1^ and a current density of 0.5 ± 0.003 A_RMS_ dm^− 2^ were needed to render all individuals (*N* = 10) immediately unconscious, as during two separate trials where field strength of 4 ± 0.02 V_RMS_ cm^− 1^ and current density of 0.4 ± 0.001 A_RMS_ dm^− 2^ were used lead to 1 fish (per trial) to be inconclusively stunned, thus leading to 80% success rate (Table 1). However, this short shock only stunned the zebrafish transiently, as all individuals recovered within the 30 min observation period. When the shock duration in the experimental tank was extended to 30 s, it resulted in euthanasia for all individuals (*N* = 10, number of trials = 2) as no signs of recovery were observed within the 30 min observation period (Table 1).

### Application of portable electrodes to euthanise zebrafish in standard housing aquaria

In the 3.5 L housing aquarium with 10 fish, a 30 s electrical shock using the same parameters as in the experimental tank (i.e. an electrical field strength of 5 ± 0.02 V_RMS_ cm^− 1^ and a current density of 0.5 ± 0.003 A_RMS_ dm^− 2^) successfully euthanised all fish (*N* = 110, number of trials = 11) with no signs of recovery. However, when fish density was increased to the maximum housing capacity of 15 fish in the 3.5 L aquarium, the success rate decreased from 100 to 93% (i.e., one out of the fifteen fish recovered in 1.3 min). At maximum housing capacity in the 3.5 L aquarium, the electrical field strength and current density needed to be increased to 6 ± 0.1 V_RMS_ cm^− 1^ and 0.7 ± 0.009 A_RMS_ dm^− 2^ respectively, to achieve 100% success rate (*N* = 75, number of trials = 5) with a 30 s shock (Table 1).

In the 8 L housing aquarium with 10 fish, using the electrical parameters from the experimental tank (i.e., an electrical field strength of 5 ± 0.02 V_RMS_ cm^− 1^ and a current density of 0.5 ± 0.003 A_RMS_ dm^− 2^) resulted in a partial success rate of 90%, as four out of the forty fish recovered (Table 1). Although the first two conducted trials with these parameters and aquarium size were successful, in the third trial two fish recovered in 2.3 and 13.0 min and in the fourth trial two fish recovered in 0.9 and 1.3 min. An electrical field strength of 6 ± 0.03 V_RMS_ cm^− 1^ and a current density of 0.7 ± 0.07 A_RMS_ dm^− 2^ was needed to achieve 100% success rate (*N* = 80, number of trials = 2) with a 30 s shock (Table 1). Yet when the fish density was increased to the maximum housing capacity of 40 fish, the success rate decreased to 98% (i.e. 2 out of 80 fish recovered in 6.4 and 0.4 min in separate trials). At maximum housing capacity in the 8 L aquarium, the electrical field strength and current density needed to be further increased to 7 ± 0.02 V_RMS_ cm^− 1^ and 0.8 ± 0.01 A_RMS_ dm^− 2^ respectively, 100% success rate (*N* = 240, number of trials = 6) to be achieved with a 30 s shock (Table 1).

While it is evident that zebrafish can be euthanised using electricity, these results open up for further investigations as they show that the efficacy of the electric shock can be contingent on both aquarium size and influenced by the fish density.

### Perspectives and conclusion

**Fig. 2:**
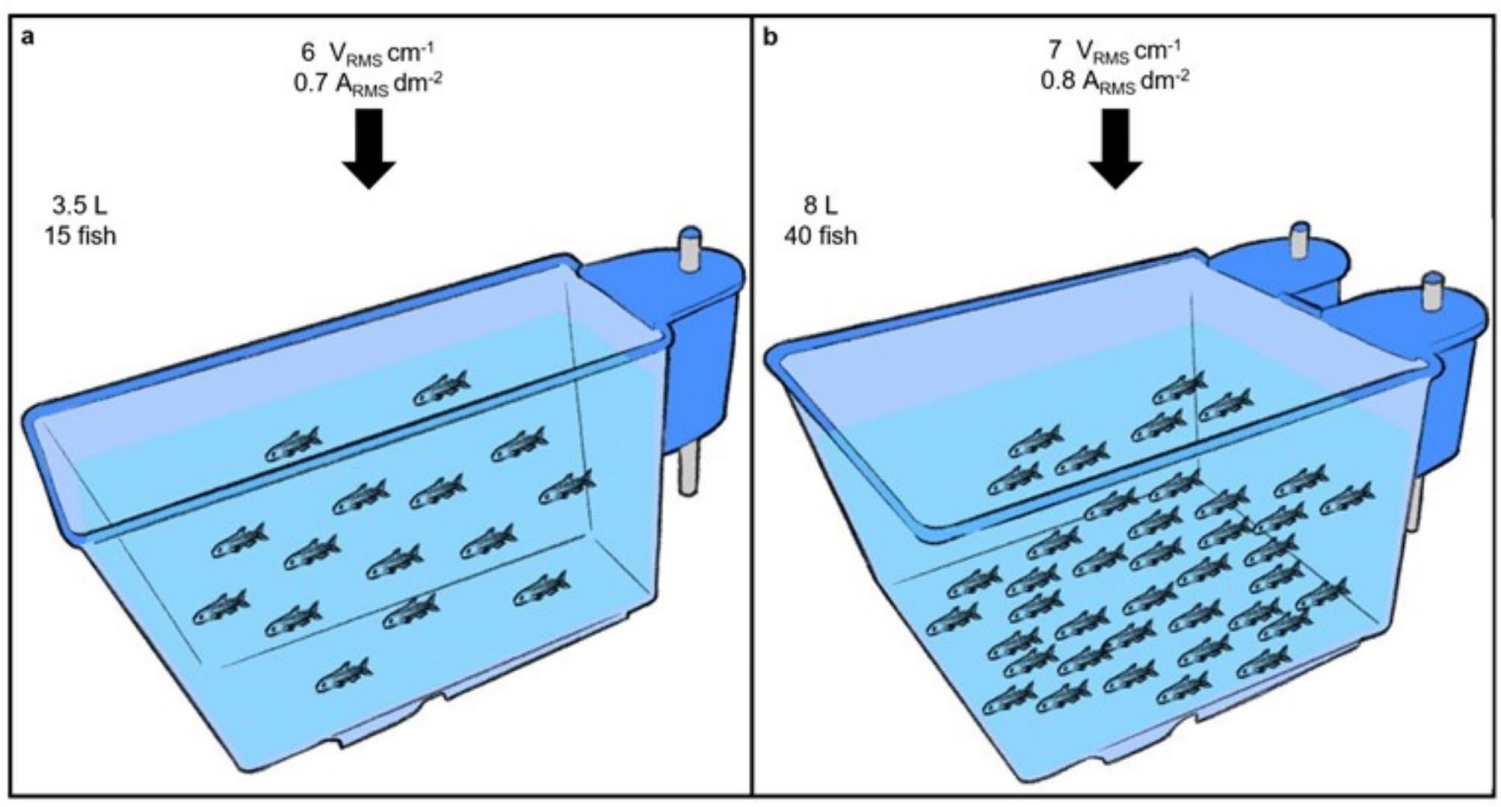
Recommended electrical parameters for in-water euthanasia of zebrafish in 3.5 L (**a**) and 8 L (**b**) Tecniplast housing aquaria. Adult zebrafish can be successfully euthanised in groups at maximum housing capacity (5 fish L^− 1^) when sufficient electrical power is applied through the aquaria at 50 Hz for 30 s in water at 28 °C with a conductivity of 800 µS cm^−1^.

To our knowledge, this is the first study to evaluate the feasibility of euthanising adult zebrafish using electrical shocks delivered directly through the water in their housing aquaria. Our results demonstrate that adult zebrafish can be euthanised in batches at their maximum housing capacity when sufficient electrical power is applied through the aquaria (Fig. 2). Therefore, our results highlights the potential for electricity as an effective method for euthanising adult zebrafish. Implementation of this method could significantly improve the welfare of zebrafish during the time of killing in laboratory settings by eliminating the aversive effects associated with current methods such as overdose of anaesthetics and live-chilling^12,13,15^.

From an animal welfare perspective, another benefit of this approach is that it allows for direct application of electrical shock to the zebrafish home tank, minimizing the need for handling the fish. With this method, the panel electrodes can be placed directly into the aquarium, allowing for batch euthanasia in their housing environment. This is particularly advantageous for zebrafish welfare, as actions like netting, air exposure, and changes in the environment can induce stress^36,37^. Additionally, euthanasia using electric shocks does not lead to any residues in the water from anaesthetic use that need to be managed. These features will also significantly reduce the workload of the personnel responsible for managing large zebrafish colonies, as well as serving the welfare of younger zebrafish since older fish may be more susceptible to diseases that could compromise all fish on the same system^7^.

The intensity, frequency and duration of the electric shocks utilized for effective euthanasia in common-sized Tecniplast aquaria, as shown in this study, are easily achievable minimum settings with a power supply typically found in animal facilities and laboratories. Consequently, using electricity as a euthanasia method holds significant potential for refinement in laboratory settings. Yet, the reasons for the observed variations in euthanasia effectiveness with different tank or aquarium sizes and population densities remain unclear and warrant further investigations. Importantly, this study determined a specific set of electrical parameters effective within our laboratory housing conditions. However, the electrical parameters required under different conditions, such as other water conductivities, temperatures, batch sizes, ages, or aquarium dimensions, might be different. For example, this method has only been validated at a conductivity of 800 µS cm^− 1^, but the conductivity of the housing water for zebrafish colonies can vary between 200 and 3000 µS cm^− 1^^38^. Furthermore, improvements to prevent fish from hiding between the aquarium wall and the electrode should be considered. For example, using thicker silicone tubing on the edges of the electrodes could mitigate this issue. An advantage with this method, however, is that the electrode panels developed in this study can be easily adapted for use with various types and dimensions of housing aquaria.

An important concern about this euthanasia technique is the operator’s safety, as in this current design it is possible for the operator to be in contact with the electric field. Therefore, we have also assessed a safety plan for our trials that can be inspected in the supplementary material to minimise any possible risks related to the operator being exposed to an electric shock. During our trials, incidents or “close calls” did not occur, but we would like to emphasise the importance of conducting a site-specific risk assessment prior to adopting this method.

Due to the small size of zebrafish, the use of behavioural assessments to evaluate the effectiveness of electric shock was employed in this study. However, future investigations of this method would benefit from neurophysiological assessments of consciousness to confirm our results about both immediate stun effects, as well as euthanasia. This is because interpretations based solely on behavioural assessments and visual indicators of consciousness may underestimate the time it takes for a fish to lose consciousness and overestimate the time for recovery^39,40^. The most reliable approach to evaluate consciousness in fish involve measuring neurophysiological activity via electroencephalogram (EEG)^39–43^. Currently, such evaluations are not feasible in small fish species, but advancements in technology and miniaturization may make this possible in the future, even for small species like zebrafish.

Safeguarding animal welfare at the time of killing by providing a humane death is a legal requirement in several countries. Common methods, such as anaesthesia overdose and live chilling require handling and pose a risk of exposing the fish to a stressful situation. Utilizing electric shock for euthanasia of adult zebrafish circumvents these issues, offering a significant refinement under the 3Rs (Replacement, Reduction, and Refinement) ethical framework^44^. Given the extensive use of zebrafish in scientific research, this new technique holds the potential to enhance the welfare of a considerable number of animals globally.

## Electronic supplementary material

Below is the link to the electronic supplementary material.

Supplementary Material 1

Supplementary Material 2

## Data Availability

The data that support the findings of this study are openly available in http://doi.org10.6084/m9.figshare.26653054.
