# Supplementary material for: Novel euthanasia technique for zebrafish using electric shock in standard group housing aquaria
Source: Sci Rep. 2025 Jan 23;15:3011. doi: 10.1038/s41598-025-87540-4 (PMC11758068; doi:10.1038/s41598-025-87540-4)
Supplement: Supplementary file 1 — Supplementary Material 1 [file 41598_2025_87540_MOESM1_ESM.pdf]

SUPPLEMENTARY MATERIAL

NOVEL EUTHANASIA TECHNIQUE FOR ZEBRAFISH: USE OF ELECTRIC SHOCK IN STANDARD GROUP HOUSING AQUARIA

Ulla Saarinen[1]*, Erika Sundell[1], Lynne Sneddon[2], and Albin Gräns[1]

[1]Department of Applied Animal Science and Welfare, Swedish University of Agricultural Sciences.

[2]Department of Biological & Environmental Sciences, University of Gothenburg.

Safety Plan

Here we detail our risk assessment and safety plan, which may help users who adopt this method to understand the potential risks and assist in the creation of a users' site-specific risk assessment.

To mitigate risks associated with electricity, we implemented safety protocols. The protocol required the presence of at least two operators during the application of the electric shock. Any nearby individuals were informed whenever the electric field was activated. The procedures were not performed at the same time as other experiments within the room to prevent potential risk exposure. The electrical device was turned on as soon as possible after fish were placed in the tank/aquaria and turned off and unplugged immediately after the electrical exposure took place. The setup was always examined before turning on the electric field to avoid hazards caused by faulty equipment. As the experimental tank contains water, the surroundings of the tank and the floor were kept as dry as possible.

During the electric exposure, one operator applied the electricity while the second operator monitored the experimental tank and aquaria. The experimenter activating the electric field communicated clearly before applying electricity, as well as when the electrical application ceased and the device was turned off. The second experimenter observed the experimental tank and aquaria from a safe distance of at least 0.5 meters, refraining from touching any surfaces. Physical contact with the experimental tank, electrode panels or the water inside the tank during the exposure was strictly prohibited. Experimenters wore rubber boots for insulation from the ground and avoided wearing conductive material like metal. A comprehensive risk assessment was conducted prior to trials, addressing general risks and specific protocols for the experimental location. Emergency procedures were established to address potential incidents such as electrical shock or fire.

[Movie]

SMovie 1. Video of a trial where zebrafish (AB strain) are shocked for 30 s in the 8 L Techniplast aquarium at maximum capacity (N=40; i.e., 5 fish L^{-1}). In this trial, a field strength of 7 V_{RMS} cm^{-1} and current density of 0.8 A_{RMS} dm^{-2} was applied. The trial was determined to be successful as no signs or recovery was observed within the 30 min observation period post electric shock.
